# A Novel CDX2 Isoform Regulates Alternative Splicing

**DOI:** 10.1371/journal.pone.0104293

**Published:** 2014-08-07

**Authors:** Matthew E. Witek, Adam E. Snook, Jieru E. Lin, Erik S. Blomain, Bo Xiang, Michael Magee, Scott A. Waldman

**Affiliations:** 1 Department of Radiation Oncology, Kimmel Cancer Center & Jefferson Medical College, Thomas Jefferson University, Philadelphia, Pennsylvania, United States of America; 2 Department of Pharmacology and Experimental Therapeutics, Thomas Jefferson University, Philadelphia, Pennsylvania, United States of America; University of Kansas School of Medicine, United States of America

## Abstract

Gene expression is a dynamic and coordinated process coupling transcription with pre-mRNA processing. This regulation enables tissue-specific transcription factors to induce expression of specific transcripts that are subsequently amplified by alternative splicing allowing for increased proteome complexity and functional diversity. The intestine-specific transcription factor CDX2 regulates development and maintenance of the intestinal epithelium by inducing expression of genes characteristic of the mature enterocyte phenotype. Here, sequence analysis of CDX2 mRNA from colonic mucosa-derived tissues revealed an alternatively spliced transcript (CDX2/AS) that encodes a protein with a truncated homeodomain and a novel carboxy-terminal domain enriched in serine and arginine residues (RS domain). CDX2 and CDX2/AS exhibited distinct nuclear expression patterns with minimal areas of co-localization. CDX2/AS did not activate the CDX2-dependent promoter of guanylyl cyclase C nor inhibit transcriptional activity of CDX2. Unlike CDX2, CDX2/AS co-localized with the putative splicing factors ASF/SF2 and SC35. CDX2/AS altered splicing patterns of CD44v5 and Tra2-β1 minigenes in Lovo colon cancer cells independent of CDX2 expression. These data demonstrate unique dual functions of the CDX2 gene enabling it to regulate gene expression through both transcription (CDX2) and pre-mRNA processing (CDX2/AS).

## Introduction

Gene expression is a highly dynamic and efficient process that couples transcription with pre-mRNA processing [1]. The functional union of gene transcription with processing of nascent transcripts occurs in a spatially organized manner in the nucleus. Activation of RNA polymerase II (RNAP II) occurs in conjunction with binding of *trans*-proteins to *cis*-elements in gene promoters and recruitment of members of the SR protein family of splicing factors [2], [3]. Regulation of transcription occurs through DNA modifications and changes in transcription factor expression, activity, and localization. In addition to these generalized processes, transcription factor expression and activity is also subjected to tissue-specific mechanisms of regulation [4]–[6]. This process enables expression of restricted sets of tissue-specific transcripts [4]–[6]. Subsequently, these transcripts are processed by ubiquitously expressed splicing factors to generate alternative transcripts, thereby increasing proteome complexity and ensuring functional diversity.

Unlike the well-defined role of *trans*-acting proteins in regulating tissue-specific gene expression, the generation of cell type- and tissue-specific proteomes has only recently been linked to changes in splicing factor expression and activity [7] [8]. Notwithstanding regulation through changes in expression and activity, few tissue-specific splicing factors have been identified. Most notably, NOVA1 and NOVA2 neuron-specific KH-type RNA binding proteins regulate splicing of transcripts encoding numerous synaptic proteins [9]. Despite the paucity of other examples, the importance of NOVA1 and NOVA2 in modulating neuronal gene expression underscores the possibility that lineage-specific splicing factors regulate gene expression in other tissues. Further, genes encoding multiple proteins that possess disparate functions in transcription and splicing may enhance lineage-specific gene expression in regulating specialized cell functions. This model is best represented by tissue-restricted expression of two isoforms of the Wilms’ tumor gene WT1, WT(-KTS) and WT1(+KTS), which regulate gene transcription and RNA processing, respectively [10] [11].

Development and maintenance of a functional intestinal epithelium is dependent on complex temporal and spatial regulation of gene expression and the resulting proteome. This process is regulated in part by a set of intestine-specific proteins, including the transcription factor CDX2. CDX2, a *caudal*-related intestine-specific transcription factor, plays a central role in regulating the development and maintenance of the intestinal epithelium canonically through promoter binding and activation of target genes, which largely comprise the mature enterocyte phenotype. These include proteins such as guanylyl cyclase C (GUCY2C), LI-cadherin, sucrase isomaltase, and lactase. Expression of this gene signature influences homeostatic processes in intestinal epithelial cells including proliferation and differentiation [12]–[18]. Here, we identify a novel CDX2 transcript (CDX2/AS) resulting from alternative splicing that is expressed concomitantly with wild type CDX2 in normal colonic epithelial cells, colonic tumors, and colon cancer-derived cell lines. CDX2 and CDX2/AS exhibited diffuse and focal nuclear localization patterns, respectively. Further, only minimal areas of co-localization between the two isoforms were observed. CDX2/AS lacks the ability to induce transcription of a CDX2-dependent promoter and does not exhibit dominant negative activity with respect to CDX2. Interestingly, CDX2/AS, but not CDX2, exhibits homology with SR and SR-like proteins and co-localizes with the spliceosome proteins ASF/SF2 and SC35. Further, CDX2/AS, but not CDX2, modulates the splicing pattern of the exogenous CD44v5 and Tra2β-1 minigene transcripts in Lovo human colorectal cancer cells independently of CDX2 expression. Thus, CDX2 is the first gene identified that encodes intestine-specific proteins that independently regulate transcription (CDX2) and alternative splicing (CDX2/AS).

## Materials and Methods

### Ethics statement

Animal protocols were approved by the Thomas Jefferson University Institutional Animal Care and Use Committee.

### Cell culture and human specimens

The Lovo colorectal cancer cell line (American Type Culture Collection Manassas, VA, USA) that underwent targeted deletion of the CDX2 locus to produce Lovo^CDX2−/−^ was obtained from Dr. DT Dang (Department of Internal Medicine, Division of Hematology/Oncology, University of Michigan, Ann Arbor, MI, USA). The human embryonic kidney cell line 293T was obtained from Dr. Laurence Eisenlohr (Thomas Jefferson University Hospital, Philadelphia, PA). HCT1116, HT29, Lovo, RKO, SW480 and T84 human colorectal cancer cells were obtained from American Type Culture Collection (Manassas, VA, USA). Cells were maintained at 37°C under 5% CO_2_ in DMEM or DMEM/F12 (Mediatech, Inc., Manassas, VA, USA) supplemented with 10% fetal bovine serum (Thermo Scientific Hyclone, Logan, UT, USA) and penicillin with streptomycin (Life Technologies, Grand Island, NY, USA). Colonic mucosal specimens were obtained from the Department of Pathology, Anatomy and Cell Biology of Thomas Jefferson University Hospital (Philadelphia, PA) under an Institutional Review Board–approved protocol (control no. 98.0614). Total RNA was extracted from human specimens with Tri-Reagent (Molecular Research Center, Inc., Cincinnati, OH, USA) and from cell lines with RNeasy columns (Qiagen, Valencia, CA, USA). Human tissues and cell culture specimens were treated with Laemmli buffer to generate whole cell lysates. All protein preparations were supplemented with protease and phosphatase inhibitors (Roche Diagnostics GmbH, Manhein, Germany).

### Sequence analysis of CDX2 and CDX2/AS

CDX2 and CDX2/AS cDNA were generated from RNA isolated from intestinal specimens by reverse transcription at 50°C for 30 minutes then 95°C for 5 minutes using primer hCDX2RT (5′-GCTCAGCCTGGAATTGC-3′). Amplification reactions were carried out for 30 cycles at 94°C for 20 seconds, 62°C for 20 seconds, and 72°C for 45 seconds using forward primer hCDX2F1 (5′-ATGTACGTGAGCTACCTCCTGGAC-3′) and reverse primer hCDX2RT. PCR products were ligated into the pGEM-T Easy Vector (Promega, Madison, WI, USA) and sequenced at the Kimmel Cancer Institute Nucleic Acid Facility (Thomas Jefferson University, Philadelphia, Pennsylvania).

### Plasmid generation, cell transfection, and retroviral transduction

CDX2 and CDX2/AS inserts from pGEM-T-CDX2 and pGEM-T-CDX2/AS were subcloned into pcDNA3.1/NT-GFP-TOPO (Life Technologies) and the murine retroviral vector pMSCV2.2 using restriction sites BglII/HindIII. Carboxy-terminal Flag (DYKDDDDK) and hexa-histidine epitodes were added to CDX2 and CDX2/AS, respectively. For control, EGFP was cloned into pMSCV2.2. Generation of the luciferase reporter constructs containing the proximal GUCY2C promoter (GUCY2C-Luc) and proximal GUCY2C promoter containing a mutated CDX2 binding site GUCY2C (GUCY2C_CDX2mut_-Luc) were generated as described previously [15]. *Renilla* luciferase control reporter, pRL-TK, driven by a viral thymidine kinase promoter was commercially available (Promega). Plasmids encoding EGFP-ASF/SF2, CD44v5, and Tra2-β1 minigene were obtained from Dr. Brian Morris (Basic Clinical Genomics Laboratory, School of Medical Sciences and Bosch Institute, The University of Sydney, NSW 2006, Australia). Cells were transfected with Fugene HD Transfection Reagent at 3 µL/µg DNA (Promega). For retroviral transduction, 2×10^5^ 293T cells plated in 60 mm dishes were transfected with 2 µg of packing plasmid pCL-ampho (IMGENEX, San Diego CA, USA) and 2 µg of a pMSCV2.2 vector containing either EGFP, CDX2-Flag, or CDX2/AS-His. Twenty-four hours after transfection, media was replaced on transfected 293T cells, and HT29 and SW480 cells were plated at 2×10^5^ in separate 60 mm dishes. After 24 h, virus-containing media from transfected 293T cells was filter through a 0.45 µm filter (Millipore Ireland Ltd, Tullagreen, Carrigtwohill IRL), supplemented with polybrene, and added to HT29 and SW480 cells, which were incubated with virus containing media for 6 h on two consecutive days. Transduced cells were selected and maintained in complete medium and 2.5 µg/mL of puromycin (Life Technologies).

### Reporter assays

Transcriptional activity of CDX2 and CDX2/AS was examined using the luciferase reporter construct pGL-3-Basic (negative control; Promega) and GUCY2C-Luc. During plating of 1×10^5^ 293T cells in 24-well plates, cells were co-transfected with 0.5 µg of either pGL-3-Basic or GUCY2C-Luc reporter constructs with 0.005 µg of pRL-TK and 0.5 µg of either expression vector pMSCV-EGFP, MSCV-CDX2-Flag, or MSCV-CDX2/AS-His using Fugene HD transfection reagent (Roche Diagnostics). Two days after transfection, cells were harvested and assayed using the protocol and materials in the Dual-Luciferase Reporter Assay system (Promega). Luminescence was measured with a TD-20/20 luminometer (Turner Designs, Sunnyvale, CA). Luciferase expression was normalized to pRL-TK expression for standardization of transfection efficiency (ratio). Promoter activity was normalized by subtracting the pGL3 control vector ratio from the respective promoter fragment–containing reporter vector ratio. In dominant negative experiments, a total of 1 µg of expression vector was used: 0.5 µg of MSCV-CDX2 and MSCV-CDX2/AS were used together while 0.5 µg of MSCV-EGFP with either MSCV-CDX2 and MSCV-CDX2/AS to keep the 1 µg of total DNA consistent. GUCY2C_CDX2mut_-Luc, which lacks the CDX2 binding site, was used a negative control [15] and pRL-TK as above. Experiments were performed five times in triplicate. Student’s *t*-test was performed to assess statistical significance.

### Splicing assay

Lovo^CDX2−/−^ cells were co-transfected with 2 µg of either or CD44v5 or Tra2β-1 minigenes with either 3 µg of pMSCV-EGFP or pMSCV-CDX2/AS-His and when noted, 3 µg of pMSCV-CDX2-Flag. Empty pMSCV plasmid was added to ensure equal amounts of transfected DNA. RNA was isolated with RNAeasy columns (Qiagen) after 36 hours of transfection. Reverse transcription and PCR amplification of CD44v5 and of Tra2β-1 transcripts was performed as previously described [19]. PCR products were resolved on 1% agarose gel and visualized with ethidium bromide using the Kodak 400R Image Stage. Band intensities were quantified with Image J (Java).

### Western blot and fluorescent immunohistochemistry

Protein samples were separated on 12% or 8–16% SDS-polyacrylamide gradient gels under reducing conditions and transferred to nitrocellulose using the iBlot Gel Transfer System (Life Technologies). After transfer, membranes were incubated in 5% non-fat dry milk in Tris-buffered saline containing 0.1% Tween-20 (TBS-T) to block non-specific protein binding. After blocking, membranes were incubated overnight at 4°C with primary antibodies in TBS-T containing either 5% non-fat dry milk or 5% bovine serum albumin. Primary antibodies used in Western blot analysis included: rabbit anti-CDX2_ACT_ that was raised against a peptide corresponding to the amino-terminal activation domain, rabbit anti-α-tubulin (Santa Cruz Biotechnology, Santa Cruz, CA), and rabbit-anti-CDX2/AS that was generated against a peptide corresponding to the entire RS domain of CDX2/AS. After overnight incubation, membranes were washed three times in TBS-T then incubated in horseradish peroxidase–conjugated bovine anti-rabbit antibody (Santa Cruz Biotechnology) in TBS-T and 5% non-fat dry milk at room temperature for 1 hour. Membranes were washed three times and protein antibody complexes were incubated in SuperSignal West Pico Chemiluminescent Substrate (Thermo Scientific) and visualized by chemiluminescent detection using the Kodak IS4000R system. Fluorescent immunohistochemistry was performed with GFP tagged CDX2, CDX2/AS, and ASF/SF2, primary antibodies: mouse anti-SC35 (Abcam, Cambridge, MA, USA product #ab11826), rabbit anti-CDX2_ACT_, rabbit anti-hexa-histidine (Cell Signaling, Danver, MA, product #2365), and rabbit anti-FLAG (Cell Signaling, product #2368), and secondary antibodies: goat anti-rabbit FITC (Santa Cruz product #2012) and TRITC (Santa Cruz product #2780), and goat anti-mouse FITC (Santa Cruz product #2781). Nuclei were stained with DAPI.

### *In vivo* splicing assay

T84 cells (5×10^6^) were injected into the flanks of immnodeficient mice (NOD.Cg-Prkdc^scid^ Il2rg^tm1Wjl^/SzJ, The Jackson Laboratory, Bar Harbor, Maine, USA) in a 50% matrigel solution (BD Biosciences, San Jose, CA, USA) and grown for 2 weeks. Mice were anesthetized with isoflurane and 10 µg of the Tra2β-1 minigene and 2 µg of either MSCV-EGFP or MSCV-CDX2/AS-His plasmid in 50 µl water were delivered into tumors. DNA delivery was followed by 10 electric pulses (field strength = 100 V/cm; pulse length = 20 ms; pulse interval = 1s) delivered by an ECM830 Square Wave Electroporation System (BTX Harvard Apparatus, Holliston, MA, USA). Mice were euthanized 3 days after electroporation. Tumors were excised and flash frozen in liquid nitrogen. RNA was then obtained via a modified RNeasy kit (Qiagen) extraction protocol. Briefly, frozen samples were thawed into RLT lysis buffer according to manufacturer’s specifications and homogenized using a mini-bead beater apparatus (Biospec Products, Bartlesville, OK, USA) and Zirconia/Silica beads (Biospec Products). The resulting homogenate was pelleted for 5 minutes at 1000×g and the supernatant was used to complete the extraction protocol. RNA samples were quality controlled for concentration and purity using a Nanodrop ND-1000 Spectrophotometer. Tra2β-1 RT-PCR amplification, visualization, and quantification is described above (Splicing assay).

## Results

### CDX2 is alternatively spliced

**Figure 1:**
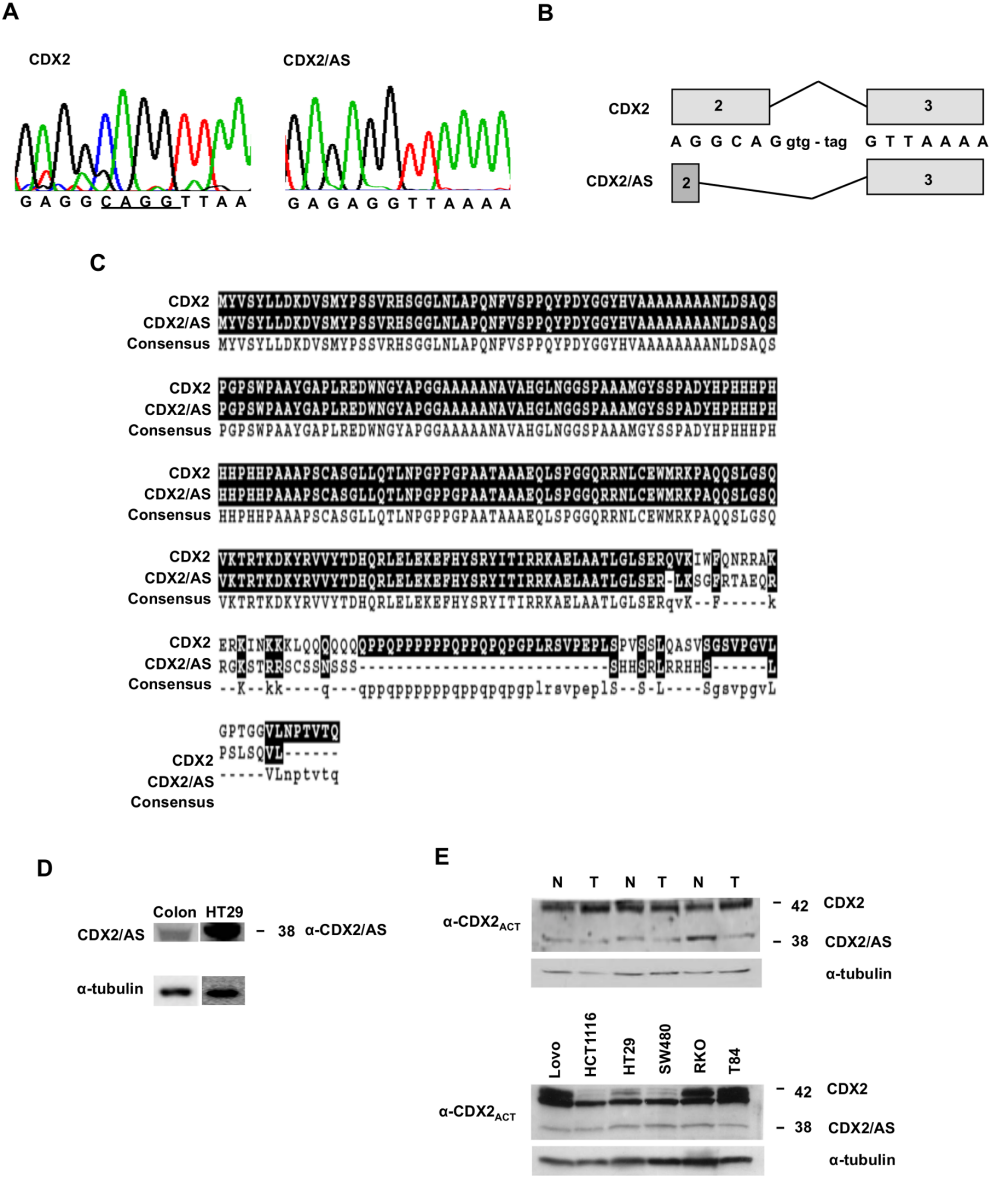
CDX2 is alternatively spliced. (A) Sequence analysis of CDX2 mRNA isolated from normal colonic epithelial cells revealed wild type mRNA and a variant transcript (CDX2/AS) lacking the bases CAGG. (B) Alternative splicing of CDX2 occurs via utilization of the non-canonical splice donor sequence GC located at the 3′ region of exon 2. (C) The alternatively induced frameshift results in deletion of the carboxy-terminal 85 bases in the wild type protein that are replaced by a novel 45 domain enriched in serine and arginine residues. (D) Western blot analysis of whole cell lysates from a normal colonic epithelium and from the HT29 colorectal cancer cell line blotted with anti-CDX2/AS revealed a band at 38 kD. (E) Western blot analysis of whole cell lysates from normal adjacent colonic epithelium (N) and colon adenocarcinomas (T) as well as whole cell lysates from various colon cancer cell lines blotted with antibody CDX2_ACT_ that recognizes the amino-terminal domain of CDX2 and CDX2/AS revealed two bands at 42 and 38 kD representing CDX2 and CDX2 kD representing CDX2 and CDX2/AS, respectively.

We sequenced full-length CDX2 cDNA clones generated by RT-PCR using RNA isolated from normal human colonic mucosa and identified two transcripts, wild type CDX2 and a variant transcript (CDX2/AS) lacking the four bases CAGG (Fig. 1A) (Genbank ID: KJ081251). Sequence analysis of the genomic CDX2 region corresponding to the four base pair deletion revealed an intact gene (data not shown). Given these findings, we attributed the generation of CDX2/AS to alternative splicing in which the non-canonical 5′ splice donor sequence, GC, located at the distal segment of exon 2 is utilized rather than the wild type GU donor sequence of intron 2 (Fig. 1B). The alternative splicing-induced frameshift occurs in the distal region of the homeobox binding domain and encodes a protein in which the 85 carboxyl terminal residues in the wild type protein are replaced with a novel 45 residue domain enriched in serine and arginine amino acids (Fig. 1C). Western blot analysis of whole cell lysates from normal colonic mucosa and from the HT29 colorectal cancer cell line using polyclonal antisera to the unique carboxy-terminal domain of CDX2/AS revealed a 38 kD band representing the alternatively spliced protein (Fig. 1D). Using an antibody recognizing the common amino-terminal activation domain, proteins at 42 kD (CDX2) and 38 kD (CDX2/AS) were identified in whole cell lysates from human colonic tumors, normal adjacent colonic mucosa, and several colon cancer cell lines (Fig. 1E).

### CDX2 and CDX2/AS exhibit distinct patterns of nuclear localization

**Figure 2:**
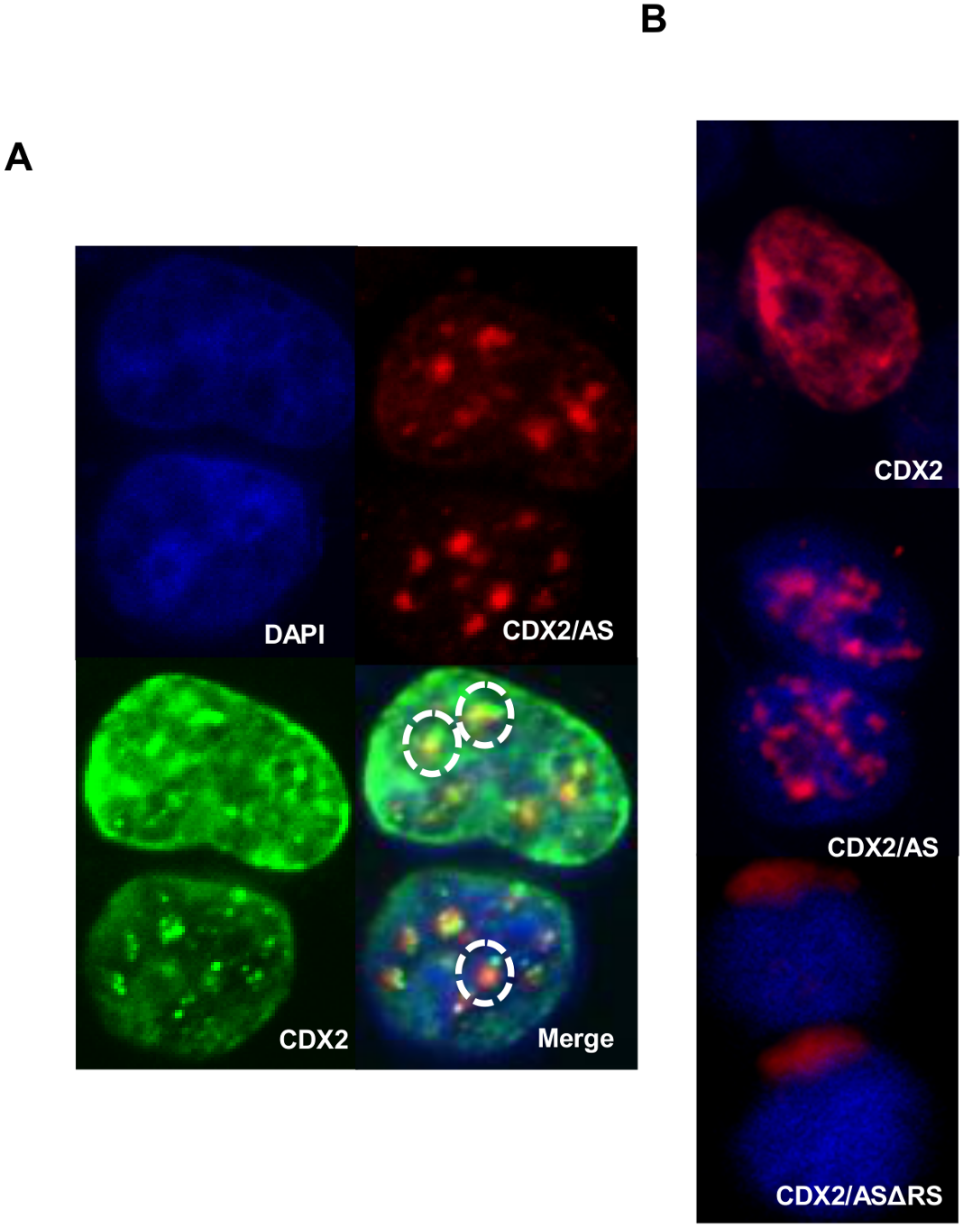
Focal nuclear localization of CDX2/AS is RS-domain dependent. (A) F-IHC analysis of 293T cells transiently transfected with MSCV-CDX2-Flag or MSCV-CDX2/AS-His revealed diffuse and focal nuclear localization patterns for CDX2 and CDX2/AS, respectively, with minimal areas of overlap (dotted circles). (B) F-IHC analysis of 293T cells transiently transfected with MSCV-CDX2-Flag, MSCV-CDX2/AS-His revealed nuclear localization whereas MSCV-CDX2/ASΔRS-His, a mutant CDX2/AS protein lacking bases distal to the alternatively induced frameshift, resulted in peri-nuclear localization.

We examined subcellular localization of CDX2 and CDX2/AS in 293T cells transiently transfected with pMSCV-CDX2-Flag and/or pMSCV-CDX2/AS-His by fluorescent immunohistochemistry (F-IHC). Although both proteins localized specifically to the nucleus, CDX2 exhibited a diffuse localization pattern in contrast to the accumulation of CDX2/AS in nuclear foci with minimal areas of co-localization with CDX2 (Fig. 2A). To determine the role of the RS domain of CDX2/AS in its subcellular localization, we performed F-IHC studies on 293T cells transiently transfected with a mutant protein (pMSCV-CDX2/ASΔRS-His) containing only bases proximal to the alternative splicing-induced frameshift. Interestingly, deletion of the RS domain resulted in perinuclear sequestration of the protein suggesting importance of these amino acids in protein stability and trafficking to the nucleus (Fig. 2B).

### CDX2/AS lacks transcriptional activity and does not function as a dominant-negative protein of CDX2

**Figure 3:**
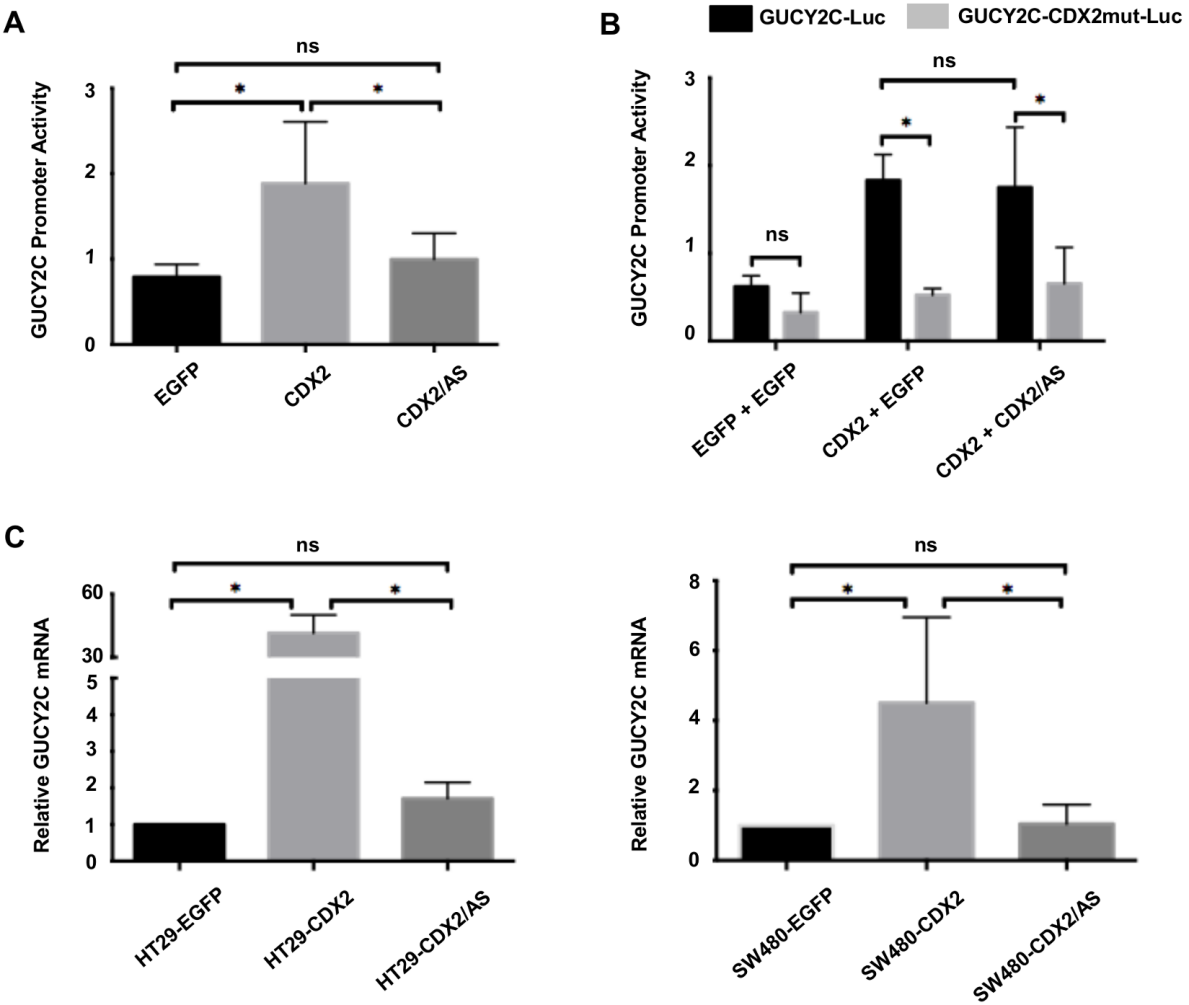
CDX2/AS lacks transcriptional activity and does not possess dominant-negative activity against CDX2. (A) 293T cells were transiently transfected with the CDX2-dependent promoter of GUCY2C (GUCY2C-Luc) along with MSCV-EGFP, MSCV-CDX2-Flag, or MSCV-CDX2/AS-His. Cells transfected with CDX2 exhibited increased luciferase activity compared to control EGFP or CDX2/AS transfected cells (p0.05). Luciferase activity between control EGFP and CDX2/AS transfected cells was similar (p = ns). (B) 293T cells were transiently transfected with either GUCY2C-Luc or GUCY2C-CDX2mut-Luc, a GUCY2C promoter with a TTT→CCC mutation in the CDX2 binding domain. Cells transfected with either GUCY2C-Luc or GUCY2C-CDX2mut-Luc plasmids were co-transfected with either 1 µg MSCV-EGFP, 0.5 µg of MSCV-EGFP and 0.5 µg of MSCV-CDX2-Flag, or 0.5 µg of MSCV-CDX2-Flag and 0.5 µg of MSCV-CDX2/AS-His. There was no difference between CDX2-induced GUCY2C-Luc activity in cells transfected with CDX2 alone or CDX2 with CDX2/AS. GUCY2C-CDX2mut-Luc was significantly different in both CDX2 alone and CDX2 and CDX2/AS transfected cells (p0.05). (C) CDX2 significantly increased expression of GUCY2C mRNA in HT29 and SW480 cells compared to EGFP expressing controls (p0.05) while GUCY2C mRNA in cells over-expressing either EGFP or CDX2/AS was similar.

Although the homology of CDX2 and CDX2/AS diverges distal to the alternative splicing-induced frameshift, the considerable structural similarities between their entire amino-terminal activation and proximal homeobox binding domains suggest that they might possess analogous functions. To explore this possibility, we compared the transcriptional activity of CDX2 with CDX2/AS in 293T cells using the CDX2-dependent luciferase reporter construct GUCY2C-Luc [15]. Cells transiently transfected with pMSCV-CDX2-Flag (1.88±0.72; p0.05) significantly increased activity of the GUCY2C-Luc reporter compared to control pMSCV-EGFP (0.79±0.15) while pMSCV-CDX2/AS-His (1.0±0.31; p = ns) was similar to control suggesting CDX2/AS does not possess the transcriptional activity inherent to CDX2 (Fig. 3A). Given the lack of transcriptional activity of CDX2/AS and the well-established role of splice variants in regulating the function of their wild type counterparts [20–[22], we next investigated the potential for CDX2/AS to influence the transcriptional activity of CDX2. We compared activation of GUCY2C-Luc in 293T cells by transiently transfecting with equal amounts of pMSCV-EGFP, pMSCV-CDX2-Flag and pMSCV-EGFP, or pMSCV-CDX2-Flag with pMSCV-CDX2/AS-His. We used the GUCY2C-CDX2mut-Luc promoter, which simulates complete inhibition of the CDX2 contribution to activation of the GUCY2C promoter, as a negative control. Here, co-transfection of pMSCV-CDX2-Flag with pMSCV-CDX2/AS-His did not significantly alter the activation of GUCY2C-Luc compared to pMSCV-CDX2-Flag alone (1.59±0.71 versus 1.75±0.69; p = ns) whereas mutation of the CDX2 binding site significantly decreased activity of the GUCY2C promoter for CDX2 alone (1.83±0.29 versus 0.52±0.07; p0.05) or in combination with CDX2/AS (1.75±0.69 versus 0.65±0.41; p0.05) suggesting that CDX2/AS does not alter the putative transcriptional activity of CDX2 (Fig. 3B).

To confirm these findings in a more physiologic setting, we used retroviral transduction to over-express either EGFP, CDX2, or CDX2/AS in HT29 and SW480 colorectal cancer cells that express endogenous CDX2 and CDX2/AS (Fig. 1E) and compared expression of endogenous GUCY2C transcripts (Fig. 3C). Similar to 293T cells, over-expression of CDX2 in HT29 and SW480 significantly increased GUCY2C mRNA levels approximately 40- and 7-fold, respectively, compared to EGFP transduced cells while over-expression of CDX2/AS had no significant effect. Taken together these data demonstrate that CDX2/AS lacks transcriptional activity and does not alter the transcriptional capacity of CDX2.

### CDX2/AS shares homology with SR and SR-like family of splicing factors

**Figure 4:**
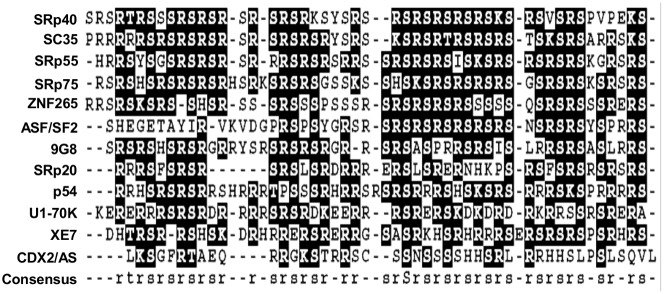
Amino-acid alignment of the RS-like domain of CDX2/AS and that of SR and SR-like proteins revealed 31% homology.

Proteins belonging to the SR and SR-like family of splicing factors typically contain an RS domain enriched in arginine and serine dipeptides and localize to punctate nuclear foci, termed nuclear speckles. Given the enrichment of arginine and serine in the carboxy-terminal domain of CDX2/AS and its focal nuclear expression, we compared the RS domain of CDX2/AS with that of other SR and SR-like splicing factors. Indeed, CDX2/AS exhibited approximately 31% homology with SR and SR-like proteins suggesting a possible role in alternative splicing (Fig. 4).

### CDX2/AS co-localizes with the putative splicing factors ASF/SF2 and SC35

**Figure 5:**
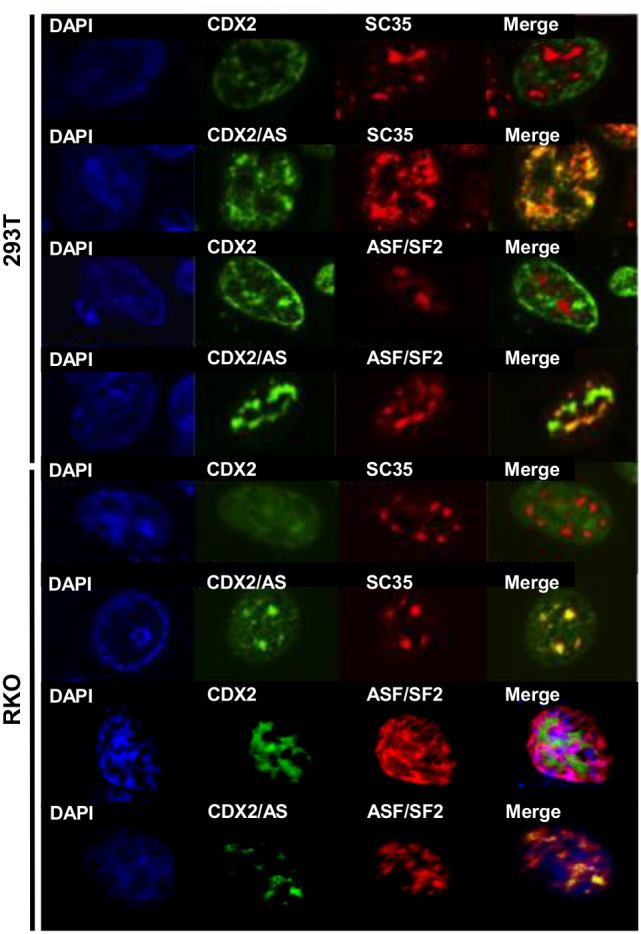
Co-localization of CDX2/AS with putative splicing factors. (A) F-IHC analysis of 293T and RKO cells transiently transfected with CDX2/AS-His and co-stained for CDX2/AS-His and either, SC35 or ASF/SF2. All proteins localized to the nucleus and merged images revealed co-localization of CDX2/AS with both SC35 and ASF/SF2. CDX2-Flag did not co-localize with either protein.

To investigate a possible role for CDX2/AS in alternative splicing, we performed F-IHC co-localization studies in transiently transfected 293T cells. Indeed, CDX2/AS co-localized with the putative SR splicing factors ASF/SF2 and SC35 whereas CDX2 did not. To confirm these findings in an endogenous system, F-IHC was performed using RKO human colorectal cells. As in 293T cells, CDX2/AS, but not CDX2, co-localized with nuclear ASF/SF2 and SC35 (Fig. 5).

### CDX2/AS influences transcriptional splice site selection

**Figure 6:**
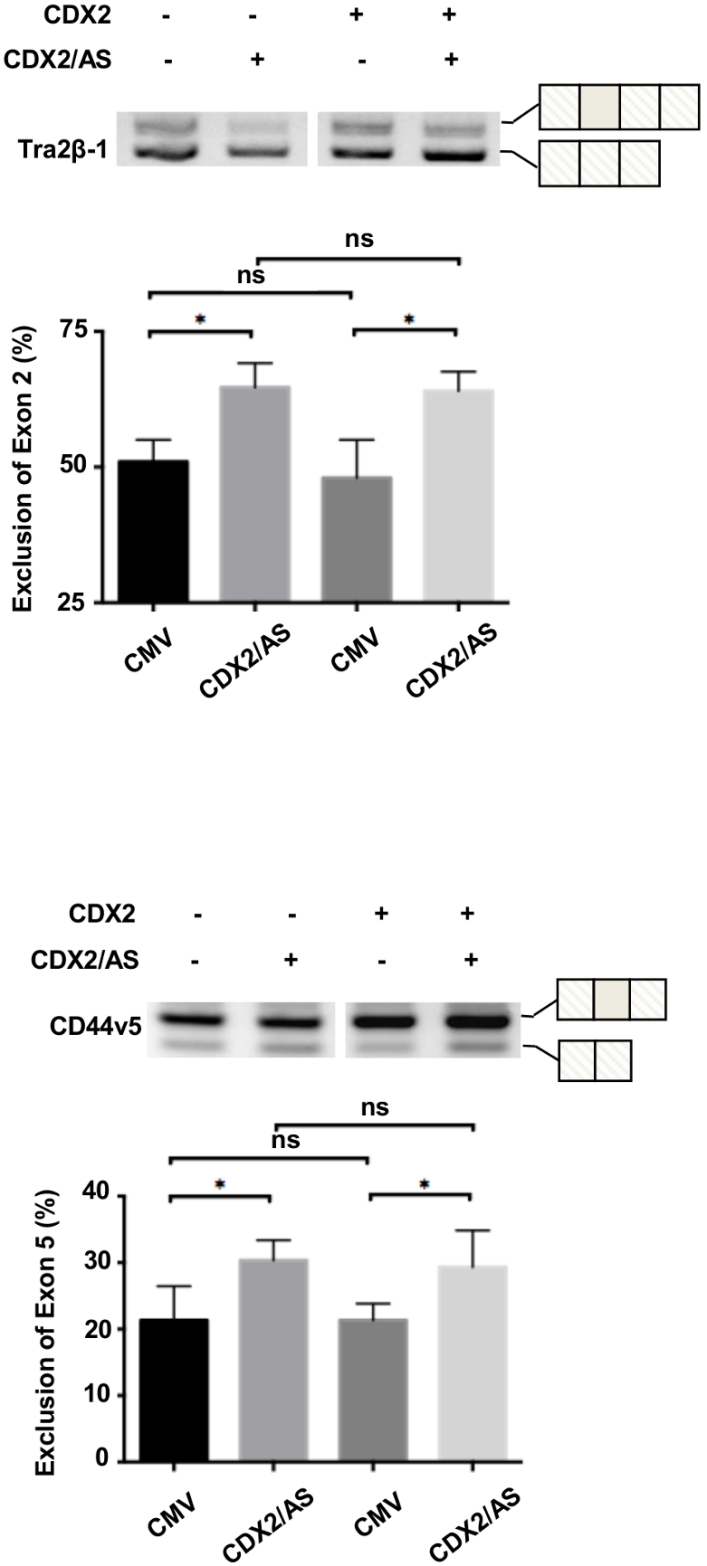
CDX2/AS influences alternative splice site selection of minigenes in 293T and Lovo^CDX2−/−^ cells. CDX2/AS regulates alternative splicing of Tra2β-1 and CD44v5 minigenes in Lovo minigenes in Lovo^CDX2−/−^ cells independent of re-introduction of CDX2 expression (p0.05). Reverse images of four independent experiments.

We next conducted splicing assays to further define a role of CDX2/AS in pre-mRNA processing. To this end, we obtained a Lovo colorectal cancer cell line devoid of CDX2 expression (Lovo^CDX2−/−^) to test the role of CDX2/AS in alternative splicing in a physiologic setting and examine the impact of CDX2 expression on CDX2/AS splicing activity [23]. We co-transfected Lovo^CDX2−/−^ cells with either a Tra2β-1 or CD44v5 minigene with or without CDX2/AS and in the absence and presence of CDX2. CDX2/AS significantly (p0.05) increased exclusion of exon 2 from Tra2β-1 and exon 5 from CD44v5 by 10%, independently of CDX2 expression, confirming the ability of CDX2/AS to alter splice site selection in colon-derived cells (Fig. 6). To evaluate these effects *in vivo*, T84 xenografts were electroporated with either MSCV-EGFP or MSCV-CDX2/AS-His and the Tra2β-1 minigene. Similar to results observed in Lovo^CDX2−/−^ cells in culture, CDX2/AS altered the splicing pattern of the minigene expressed in T84 xenografts by 10% (Fig. S1).

## Discussion

The canonical CDX2 gene comprises three exons and two introns encoding a 313 amino acid protein with an amino-terminal transactivation region and carboxy-terminal homeobox binding domain. CDX2 regulates trophectoderm integrity during embyrogenesis and postnatally the development and maintenance of the intestinal epithelium [12] [24]–29]. We report here the discovery and characterization of a novel CDX2 transcript, CDX2/AS, generated by alternative splicing, that encodes a CDX2 protein possessing the activation domain but a truncated homeobox juxtaposed distally by a novel 45 amino acid domain enriched in serine and arginine residues. Although contemporaneously expressed and localized in the nucleus, CDX2 and CDX2/AS function independently as transcription and splicing factors, respectively, establishing CDX2 as the first gene to encode intestine-specific proteins that regulate transcription and pre-mRNA processing.

In intestine, CDX2 regulates expression of genes characteristic of the differentiated enterocyte phenotype such as GUCY2C, LI-cadherin, and sucrose isomaltase [12] [15] [16]. Induction of a cassette of genes mediating intestinal differentiation, development of increased number of colonic tumors in APC^+/delta716^CDX2^+/−^ compound mutant mice, and loss of CDX2 expression in a subset of minimally differentiated colonic tumors has established CDX2 as a putative intestinal tumor suppressor [30] [31]. Paradoxically, in other studies, CDX2 increases expression of pro-proliferative genes, increases anchorage-independent growth, undergoes overexpression in colonic tumors, and even functions as a lineage-survival oncogene [23] [32]–[35]. To date, mechanisms reconciling these diametrically opposing phenotypes regulated by CDX2 have not been identified.

Homeodomain-less isoforms of other homeodomain proteins regulate the expression, localization, or activation of their associated wild type isoform. These truncated variants function as dominant negative isoforms inhibiting transcription by sequestering full-length proteins in the cytoplasm and preventing transcriptional activation of downstream targets [36]–[38]. Moreover, they interact indirectly with DNA by binding other co-factors or transcription factors activating distinct subsets of downstream targets. Thus, we explored the possibility that the paradoxical tumor suppressor and oncogenic phenotypes induced by CDX2 might reflect the generation of a previously unknown homeodomain-less variant. Indeed, sequencing of full-length CDX2 transcripts isolated from normal colonic mucosa, adjacent tumors, and colorectal cancer-derived cell lines revealed two distinct transcripts with CDX2/AS containing a frameshift-induced truncation of the homeobox domain rendering it free of transcriptional activity. The role of CDX2/AS as a dominant-negative isoform of CDX2 was explored in artificial and physiologic cell systems employing exogenous and endogenous targets of CDX2. In contrast to homeodomain-less isoforms of other homeodomain proteins, CDX2/AS did not regulate the transcriptional activity of CDX2. This result was unexpected as both proteins co-localized in the nucleus and exhibit limited co-localization providing opportunity for functional interaction. Given the many genes regulated by CDX2, CDX2/AS could regulate the expression of others not evaluated here. Similarly, CDX2/AS might induce CDX2 to regulate a previously uncharacterized set of genes that may alter cellular phenotypes. These possibilities remain to be explored.

In an attempt to determine the function of CDX2/AS, we focused on the localization of CDX2/AS to punctuate nuclear foci. Many proteins localize to distinct nuclear foci such as speckles, paraspeckles, nucleoli, Cajal bodies, GEMS, and PML bodies when observed by indirect immunofluorescence microscopy [39]. Given the enrichment in arginine and serine residues of the carboxy-terminal domain of CDX2/AS, we hypothesized that its localization was most consistent with speckled domains defined by the SR- and SR-like family of splicing factors, which contain domains enriched in arginine and serine residues. Here, we demonstrate by confocal microscopy that CDX2/AS co-localizes with the well-established SR proteins ASF/SF2 and SC35 in 293T and RKO colon cancer cells. Nuclear localization of CDX2/AS was dependent on its unique carboxy-terminal domain enriched in serine and arginine. Indeed, a mutant of CDX2/AS in which the carboxy-terminal domain enriched in serine and arginine was eliminated failed to co-localize with ASF/SF2 or SC35 in the nucleus. Supporting its roll in pre-mRNA processing, we demonstrated that CDX2/AS can change splice site selection in human colon-derived cells using exogenous minigenes. Taken together, these data strongly suggest that CDX2 encodes two proteins, one that regulates gene transcription and a novel protein that can influence gene expression by regulating pre-mRNA processing.

Whether or not CDX2/AS belongs to the SR- or SR-like families of proteins is worth considering. The RS domain of SR-and SR-like proteins is required for protein-protein interactions as well as nuclear localization. The prototypical SR splicing factors contain one or two amino-terminal RNA recognition motifs (RRMs) followed by a carboxy-terminal RS domain enriched in serine and arginine residues. Additionally, SR proteins contain signature sequences including RDAEDA, RDADDA, and SWQDLKD [40]–[45]. Homology searches of the entire CDX2/AS protein failed to reveal RRMs or signature sequences characteristic of SR proteins. These observations suggest that CDX2/AS may not be a member of the SR family of proteins but, rather, belongs to the SR-like family of splicing factors.

Interestingly, there was no clear homology in the carboxy-terminal domain of CDX2/AS with the RS domain of other proteins. Despite approximately 31% amino acid consensus with the RS domain of SR- and SR-like proteins, CDX2/AS contains only two S/R dipeptides. However, the role of the RS domain in CDX2/AS in nuclear localization is similar to that of the recently identified SR-like protein NSrp70 [46]. Moreover, perinuclear localization of the carboxy-terminally truncated CDX2/AS is identical to that seen for an RS-domain deleted mutant of the SR-like protein XE7 [19]. Taken together, these data suggest that CDX2/AS should be included in the SR-like family of splicing factors.

In the context of a single gene giving rise to alternatively spliced transcripts that produce proteins regulating transcription or splicing, CDX2 shares considerable similarity with the Wilms’ tumor gene, WT1. The WT1 transcript undergoes alternative splicing to produce several variants, most notably a transcription factor and a splicing factor. The unique ability of WT1 to regulate gene expression by encoding proteins that influence transcription and splicing in conjunction with the role of its various isoforms in normal cellular development and pathophysiologic processes suggests a similar function for CDX2/AS. For example, the ability of CDX2/AS to regulate the splicing of GUCY2C could impact numerous processes since GUCY2C is an intestinal tumor suppressor, which regulates key homeostatic functions including proliferation and the cell cycle, metabolism, differentiation, DNA damage sensing and repair, epithelial-mesenchymal interactions, and intestinal barrier function [47]–[49]. Further, the ratio of the various WT1 splice variants influences transcriptional activity and alterations contribute to pathophysiologic processes [50]–[52]. Although from our experiments, we could not demonstrate that CDX2/AS altered the activity of CDX2, if the ratio or CDX2 to X2/AS changed, the relative amount of CDX2 would be either increased or decreased. This change in expression level of CX2 could potentially increase or decrease its effective transcriptional activity.

In conclusion, we have identified and characterized a novel CDX2 splice variant, CDX2/AS, that functions independently of CDX2 as a member of the SR-like family of splicing factors. Given the significant role of CDX2 in embryogenesis, normal intestinal gene expression, and gastrointestinal tumorigenesis, our findings warrant further investigation into the contribution of CDX2/AS to these processes.

## Supporting Information

Figure S1 **CDX2/AS influences alternative splice site selection of Tra2-β1 minigene** ***in vivo*** **.** CDX2/AS significantly induced the inclusion of exon 2 in the Tra2-β1 minigene in T84 xenografts compared to control vector (p0.05). Representative image from two independent experiments.
(PDF)
