# Correction: A Novel CDX2 Isoform Regulates Alternative Splicing

**DOI:** 10.1371/journal.pone.0112164

**Published:** 2014-10-23

**Authors:** The PLOS ONE Staff

The sixth author’s name is spelled incorrectly. The correct name is: Michael S. Magee.
