# Supplementary figures and images for: A Novel CDX2 Isoform Regulates Alternative Splicing
Source: PLoS One. 2014 Aug 7;9(8):e104293. doi: 10.1371/journal.pone.0104293 (PMC4125279; doi:10.1371/journal.pone.0104293)

Supplemental Figure 1.

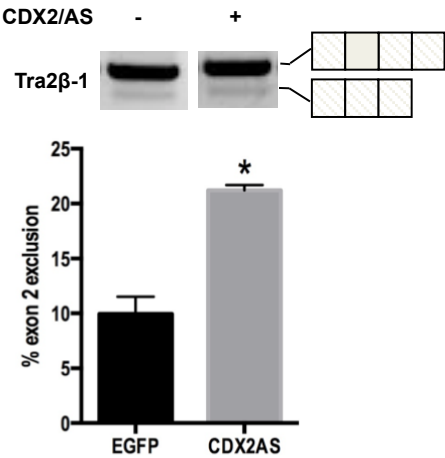

Supplement: Figure S1 — CDX2/AS influences alternative splice site selection of Tra2-β1 minigene in vivo . CDX2/AS significantly induced the inclusion of exon 2 in the Tra2-β1 minigene in T84 xenografts compared to control vector (p0.05). Representative image from two independent experiments. (PDF) [file pone.0104293.s001.pdf]
